# Gut Microbiome Alterations in Patients With Carotid Atherosclerosis

**DOI:** 10.3389/fcvm.2021.739093

**Published:** 2021-11-19

**Authors:** Jingfeng Chen, Qian Qin, Su Yan, Yang Yang, Hang Yan, Tiantian Li, Lin Wang, Xinxin Gao, Ang Li, Suying Ding

**Affiliations:** ^1^Health Management Center, The First Affiliated Hospital of Zhengzhou University, Zhengzhou, China; ^2^College of Public Health, Zhengzhou University, Zhengzhou, China; ^3^Gene Hospital of Henan, Precision Medicine Center, The First Affiliated Hospital of Zhengzhou University, Zhengzhou, China

**Keywords:** carotid atherosclerosis, gut microbiota, metabolic pathway, trimethylamine-n-oxide, lipopolysaccharide biosynthesis

## Abstract

Carotid atherosclerosis (CAS) is a reflection of systemic atherosclerosis and the main pathological processes of cardiovascular disease (CVD), namely, carotid intima–media thickening, carotid plaque formation, and carotid stenosis. Accumulating evidence indicates that the gut microbiota plays an important role in CVD and gut–brain disorders, but the associations of the composition and metabolites of the gut microbiome with CAS have not been studied comprehensively. We performed a gut microbiome genome-wide association study in 31 patients with CAS and 51 healthy controls using whole-genome shotgun sequencing. We found that several risk factors (waist circumference, body mass index, diastolic blood pressure, systolic blood pressure, fasting blood glucose, glycated hemoglobin A1c, total cholesterol, triglyceride, and low-density lipoprotein cholesterol) and inflammatory markers (white blood cell count and absolute value of neutrophils) were significantly higher in the CAS group than in the control group. In addition, 21 species and 142 pathways were enriched in the CAS group, and 10 species and 1 pathway were enriched in the control group. Specifically, *Bacteroides eggerthii, Escherichia coli*, and *Klebsiella pneumoniae* were the most abundant species in the CAS group, whereas *Parabacteroides unclassified, Prevotella copri, Bacteroides sp 3_1_19*, and *Haemophilus parainfluenzae* were the most abundant species in the control group. Finally, we found that most gut microbes and microbial pathways that were enriched in the CAS group had significant positive correlations with clinical characteristics, whereas the microbes and pathways enriched in healthy controls had significant negative correlations with clinical characteristics excluding high-density lipoprotein cholesterol. In addition, the associations between gut microbes and some microbial pathways (short-chain fatty acid, lipopolysaccharide, and menaquinol biosynthesis) were identified. Our results indicate the existence of a cyclic pathway that elevates the circulating concentrations of trimethylamine-N-oxide in patients with CAS but reduces its concentrations in healthy controls.

## Introduction

Cardiovascular disease (CVD) has become a leading cause of disability and premature mortality globally (1, 2). According to the European Cardiovascular Disease Statistics 2017, CVD causes 3.9 million deaths in Europe and more than 1.8 million deaths in the European Union, and it has become the leading cause of mortality in people younger than 75 years in Europe (3). In China, approximately 290 million people experience CVD, mainly, namely, hypertension, stroke, and coronary heart disease (4). Thus, CVD has emerged as a serious global public health problem. Atherosclerosis is regarded as the main pathological manifestation of most CVDs, and carotid atherosclerosis (CAS) is the most common and important type of atherosclerosis (5). CAS describes carotid artery stenosis or occlusive disease caused by atherosclerosis, which is divided into carotid intima–media thickening, carotid plaque formation, and carotid stenosis based on the progression of CAS. Many studies indicated that older age, male sex, smoking, diabetes, hypertension, and dyslipidemia were associated with an increased risk of CAS (2, 6, 7).

Accumulated evidence indicates that the gut microbiota plays an important role in the occurrence and development of atherosclerosis, and it has emerged as a new target for the prevention and treatment of atherosclerosis (8, 9). Previous studies revealed that the gut microbiota might indirectly affect atherosclerosis by increasing the risks of obesity and diabetes (10, 11) or directly increase the risk factors for atherosclerotic lesions by producing trimethylamine-N-oxide (TMAO) (12). Concerning TMAO, several animal experiments demonstrated that the gut microbiota can produce trimethylamine (TMA) by metabolizing dietary lecithin, and then TMA is metabolized by liver flavin monooxygenases (FMO) to produce a new independent atherosclerotic risk factor (i.e., TMAO) that can induce CVD and gut–brain disorders, such as Alzheimer's disease (13, 14). TMAO can elevate the expression of macrophage scavenger receptors, increase the expression of heat shock proteins and proinflammatory cytokines, and improve the level of plasma cholesterol to promote the occurrence and development of atherosclerotic CVD (15).

In addition, there is evidence that inhibiting TMAO production can reduce the risk of atherosclerosis. TMAO production can be regulated by probiotic or antibiotic usage, inhibition of the metabolic pathways of intestinal flora, or reduction of the dietary intake of choline and phosphatidylcholine (16). In general, there are two mechanisms known to reduce TMAO levels. In one mechanism, some bacteria (e.g., archaebiotics) can convert TMA into methane in the intestine and reduce TMAO production from TMA in the liver (17), and in the second mechanism, some antiviral agents can change the activity of flavin-containing monooxygenase 3 (FMO3) and then block or reduce the production of TMAO in the liver (18).

The existing evidence about the relationship of the gut microbiota with atherosclerosis was mainly generated in coronary atherosclerotic disease and animal experiments (16, 19). However, the lack of a cohort for the metagenomic characterization of this major group of CAS has impeded further investigations into the role of the microbiome. Although coronary atherosclerosis and CAS are mainly reflectors of atherosclerosis with some same risk factors, they have different phenotypes and disease presentations, such as coronary atherosclerosis for coronary heart disease and CAS for cerebral infarction (20). This study investigated the relationships between the gut microbiome and CAS in a cross-sectional cohort to further provide a theoretical basis to prevent and decrease the risk of CAS in China.

## Materials and Methods

### Study Population

**Figure 1:**
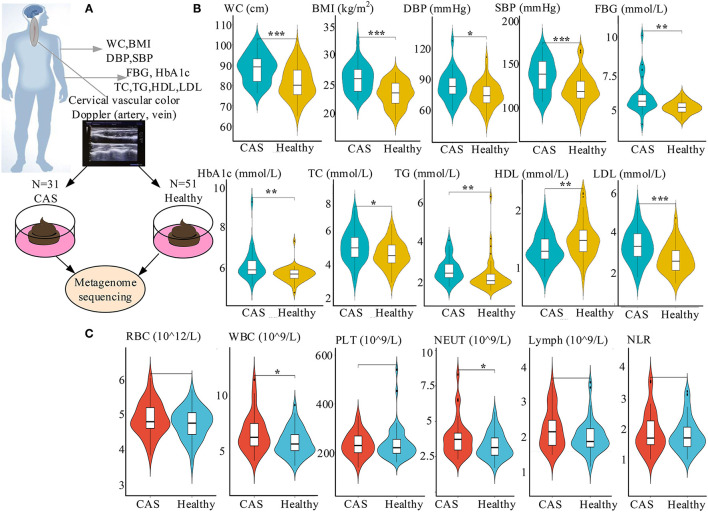
Study design and characteristics of the clinical parameters. **(A)** Study design including the cross-sectional cohort. **(B)** Comparisons of risk factors between the CAS and healthy control groups. **(C)** Comparisons of inflammatory markers between the CAS and healthy control groups. *, **, and *** denote *p* < 0.05, *p* < 0.01, and *p* < 0.001 compared with healthy controls, respectively. CAS, carotid arteriosclerosis; WC, waist circumference; BMI, body mass index; DBP, diastolic blood pressure; SBP, systolic blood pressure; FBG, fasting blood glucose; HbA1c, glycated hemoglobin A1c; TC, total cholesterol; TG, triglyceride; HDL-C, high-density lipoprotein cholesterol; LDL-C, low-density lipoprotein cholesterol; SUA, serum uric acid; RBC, red blood cell; WBC, white blood cell; PLT, platelets; NEUT, absolute value of neutrophils; Lymph, absolute value of lymphocytes; NLR, neutrophil-to-lymphocyte ratio.

In our study, 31 patients diagnosed with CAS (12 women and 19 men; mean age, 51.32 ± 6.73 years) in the Department of Health Management Center, the First Affiliated Hospital of Zhengzhou University, and 51 sex- and age-matched healthy controls (25 women and 26 men; mean age, 48.49 ± 6.17 years) were also enrolled. All participants were diagnosed by experienced ultrasound imaging physicians based on the results of Color Doppler ultrasound of neck blood vessels. Their health questionnaire and clinical data were collected prospectively through face-to-face interviews and laboratory examinations, respectively (Figure 1A). Informed consent was obtained from all subjects, and the Ethics Committee of the First Affiliated Hospital of Zhengzhou University approved this study (Number: 2018-KY-56 and 2018-KY-90).

The inclusion criterion for the CAS group was the presence of left, right, or bilateral common carotid artery or right subclavian artery atherosclerotic plaque formation. A carotid atherosclerotic plaque was defined as carotid intima–media thickness >1.4 mm or by the presence of focal wall thickening at least 50% greater than that of the surrounding vessel wall (21). The inclusion criterion for the healthy control group was the absence of abnormalities in the bilateral common carotid arteries, internal carotid artery, external carotid artery, vertebral artery, and subclavian artery. The exclusion criteria were as follows: (1) diagnosis of diabetes, hyperthyroidism, heart disease, or cancer; (2) ascending aorta widening or decreased left ventricular diastolic function; and (3) intima–media thickening of the bilateral common carotid arteries.

### Data Collection

Health questionnaire data included demographic characteristics, medical history, behavioral risk factors, and smoking and drinking habits. Subjects were not permitted to drink or eat after 11 p.m. the day before the physical examination, and their laboratory data were collected, namely, white blood cell (WBC), red blood cell (RBC), and platelet (PLT)counts; the absolute values of neutrophils (NEUT) and lymphocytes (Lymph); the neutrophil-to-lymphocyte ratio (NLR); and high-density lipoprotein cholesterol (HDL-C), low-density lipoprotein cholesterol (LDL-C), triglycerides (TG), total cholesterol (TC), fasting blood glucose (FBG), glycated hemoglobin A1c (HbA1c), and serum uric acid (SUA) levels.

Meanwhile, a Philips Affiniti 50 color Doppler ultrasound system (Philips, Tampa, FL, USA) was used to scan the neck blood vessels with a linear array probe and a frequency of 5–12 MHz. First, all subjects laid flat on the examination table to expose their necks. The carotid arteries included the bilateral common carotid arteries, internal carotid artery, external carotid artery, and vertebral artery. The examination generally started from the proximal end and progressed to the distal end to observe the clarity of the carotid artery wall layer, intima–media thickness of the wall, and formation of sclerotic plaques. The presence of carotid plaques was verified by at least two ultrasonography clinicians. In addition, the fecal samples were collected on the same day, separately packed, and then placed in a −80°C refrigerator for later shotgun metagenomic sequencing.

### DNA Extraction, DNA Library Construction, and Sequencing of Fecal Samples

According to the instructions of the manufacturer, DNA from 82 fecal samples was extracted using the MagPure Stool DNA KF kit B (Magen, Guangzhou, China). DNA was quantified using a Qubit Fluorometer, prepped with the Qubit dsDNA BR Assay Kit (Invitrogen, Carlsbad, CA, USA). Genomic DNA was broken by ultrasound to form random fragments and selected. The selected fragments were amplified and purified to obtain single-stranded circular DNA. The final library was formed after formatting and identified by quality control. The qualified library was sequenced on the BGISEQ-500 platform (BGI-Shenzhen, China).

Sequencing data were subjected to qualitative control to eliminate hybrid sequences, namely, human genome sequences, food genome sequences, and low-quality sequences. MetaPhlAn2 with the default settings was used to classify and annotate the metagenome of the sequencing library and obtain the standard relative abundance values of species at all levels (22). The NCBI.nlm.nih.gov database (2014 Edition) and HUMAnN2 (the HMP Unified Metabolic Analysis Network 2) were used to annotate the non-redundant gene set and the functional genes into Kyoto Encyclopedia of Genes and Genomes metabolic pathways and clarify the metabolic pathway (23, 24).

### Statistical Analysis

Statistical analyses were performed using the R program, version 4.0.5. Standardized statistical methods were used to analyze the demographic, laboratory, species, and pathway data. The Student's *t*-test, the Wilcoxon's rank-sum test, and the chi-squared test were the main statistical methods employed. Categorical variables were represented as numbers. Missing *n* (%) and the chi-squared test were used for association testing. Continuous variables were expressed as the mean ± SD. Differences between the groups were analyzed by normality and homogeneity testing, and *P* ≥ 0.05 indicated normal and homogenous variance. Then, parametric or non-parametric testing was performed, and *p* < 0.05 denoted statistical significance. The R function “cor.test” with the Spearman method was used to evaluate the correlations among microbial diversity, pathway diversity, and biochemical indicators. Principal coordinate analysis was performed using the R program “ade4” according to the relative abundance of microbial species or pathways. The R package “vegan” was used to calculate the Shannon index and Gini index of each sample.

## Results

### Clinical Characteristics of the Subjects

The study included a cross-sectional cohort of 31 patients with CAS and 51 healthy participants (Figure 1A). Concerning risk factors, waist circumference (WC), body mass index (BMI), diastolic blood pressure (DBP), systolic blood pressure (SBP), FBG, HbA1c, TC, TG, LDL-C, and SUA were significantly higher in the CAS group than in the control group, whereas the opposite was true for HDL-C. Similarly, regarding inflammatory markers, WBC and NEUT were significantly higher in the CAS group than in the healthy control group (Figures 1B,C; Table 1). In addition, there were no significant differences in the lifestyle habits of the two groups, such as smoking and drinking.

**Table 1:** The major demographic and serum characteristics of patients with CAS and healthy controls.

	**CAS (*n* = 31)**	**Healthy (*n* = 51)**	***P***
Gender	Female:12,Male:19	Female:25,Male:26	0.496
Age	51.32 ± 6.73	48.49 ± 6.17	0.061
WC	88.39 ± 7.01	81.53 ± 7.96	<0.001^*^
BMI	26.00 ± 2.39	23.35 ± 2.15	<0.001^*^
DBP	83.55 ± 14.26	75.33 ± 12.30	0.01^*^
SBP	136.84 ± 19.19	120.20 ± 18.22	<0.001^*^
FBG	5.93 ± 1.31	5.10 ± 0.48	0.002^*^
HbA1C	6.13 ± 0.75	5.69 ± 0.36	0.005^*^
TC	5.02 ± 0.93	4.53 ± 0.75	0.016^*^
TG	1.80 ± 0.73	1.40 ± 0.96	0.036^*^
HDL	1.33 ± 0.27	1.51 ± 0.33	0.008^*^
LDL	3.28 ± 0.83	2.66 ± 0.73	<0.001^*^
SUA	354.23 ± 72.61	297.16 ± 63.70	0.001
RBC	4.86 ± 0.49	4.74 ± 0.44	0.266
WBC	6.56 ± 1.68	5.74 ± 1.22	0.022^*^
PLT	234.13 ± 48.10	237.35 ± 67.07	0.801
NEUT	3.83 ± 1.35	3.27 ± 0.90	0.048^*^
Lymph	2.13 ± 0.56	1.90 ± 0.46	0.058
NLR	1.86 ± 0.65	1.78 ± 0.54	0.562
Smoking	No:13, Yes:2	No:20, Yes:2	1
Drinking	No:7,Yes:7	No:12,Yes:10	1

*CAS, carotid arteriosclerosis; WC, waist circumference; BMI, body mass index; DBP, diastolic blood pressure; SBP, systolic blood pressure; FBG, fasting blood glucose; HbA1c, glycated hemoglobin A1c; TC, total cholesterol; TG, triglyceride; HDL-C, high-density lipoprotein cholesterol; LDL-C, low-density lipoprotein cholesterol; SUA, serum uric acid; RBC, red blood cell; WBC, white blood cell; PLT, platelets; NEUT, absolute value of neutrophils; Lymph, absolute value of lymphocytes; NLR, neutrophil-to-lymphocyte ratio.*

* *P < 0.05*.

### Community Diversity in the CAS and Healthy Control Groups

**Figure 2:**
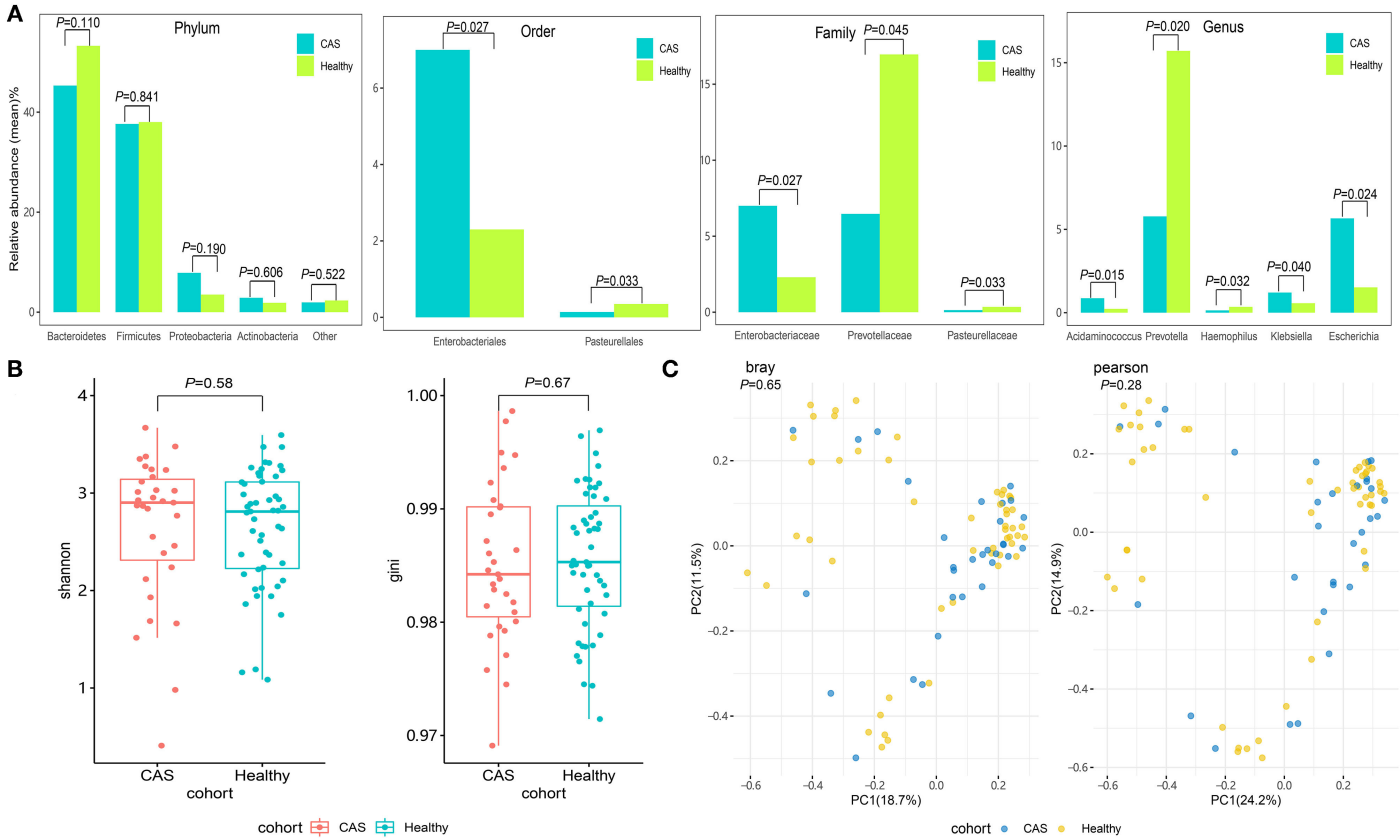
Comparison of the microbial community. **(A)** The abundant phyla, orders, families, and genera of the gut microbiota between the carotid arteriosclerosis (CAS) and healthy control groups. **(B)** Comparisons of alpha diversity as determined by the Shannon and Gini indices between the CAS and control groups. **(C)** Comparisons of beta diversity as determined by the Bray and Pearson distances between the CAS and control groups.

As presented in Figure 2A, the gut microbiota in the CAS and control groups was dominated by four abundant phyla with no significant differences between the groups. However, the abundance of Enterobacteriales and Pasteurellales at the order level; Enterobacteriaceae, Prevotellaceae, and Pasteurellaceae at the family level; and *Acidaminococcus, Escherichia, Haemophilus, Klebsiella*, and *Prevotella* at the genus level was significantly different between the groups. The abundance of Prevotellaceae, Pasteurellaceae, *Haemophilus, and Prevotella* was significantly higher in the CAS group (Supplementary Table 1). In addition, the microbiomes of patients in the CAS group at the species level had no significant differences concerning alpha diversity as determined by the Shannon and Gini indices compared with the healthy controls (Wilcoxon's rank-sum test, *p* = 0.58 and *p* = 0.67, respectively; Figure 2B). Similarly, the microbiomes of patients in the CAS group did not differ from those of healthy subjects regarding beta diversity as determined by the Bray and Pearson distances (Wilcoxon's rank-sum test, *p* = 0.65 and *p* = 0.28, respectively; Figure 2C).

### Analysis of the Microbiota Composition and Associations Between the Gut Microbiome and Clinical Characteristics

#### Analysis of the Microbiota Composition

**Figure 3:**
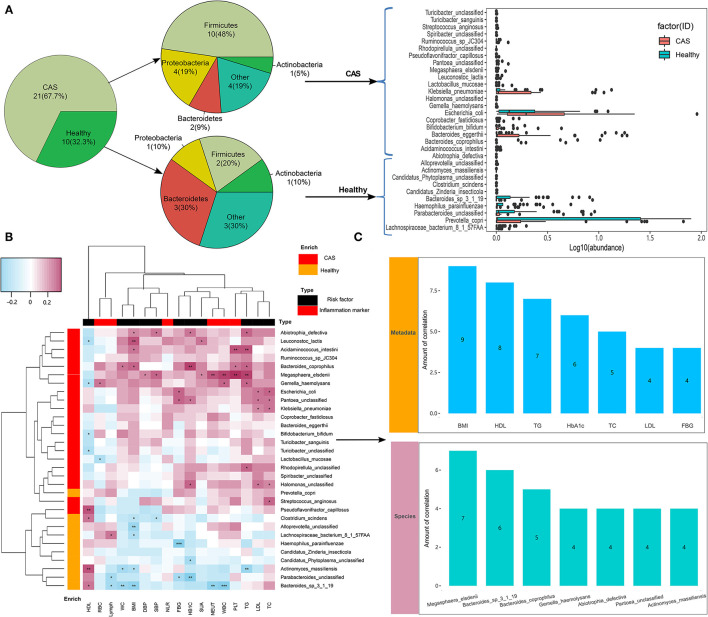
Microbiome differences between the groups and the associations with clinical characteristics. **(A)** Species with differences in abundance between the carotid arteriosclerosis (CAS) and healthy control groups. **(B)** Correlation matrix for species and clinical parameters. Pink cells denote positive correlations, whereas blue cells depict negative correlations. *, **, and *** denote *p* < 0.05, *p* < 0.01, and *p* < 0.001, respectively. **(C)** The number of significant correlations for the seven risk factors and seven species with the most frequent correlations.

We compared microbial differences at the species level between the CAS and control groups and identified 45 different species (Wilcoxon's rank-sum test, *p* < 0.05; Supplementary Table 2). After removing the species with low occurrence and abundance, 31 species with significant differences in abundance were identified (*p* < 0.05; Figure 3A), namely, 21 enriched species in the CAS group and 10 enriched species enriched in the control group (Figure 3A). In the species-level analysis, 10 species (i.e., *Abiotrophia defectiva, Acidaminococcus intestini, Gemella haemolysans, Lactobacillus mucosae, Leuconostoc lactis, Megasphaera elsdenii, Ruminococcus sp JC304, Streptococcus anginosus, Turicibacter sanguinis*, and *Turicibacter unclassified*) belonging to Firmicutes and fourspecies (i.e., *Escherichia coli, Halomonas unclassified, Klebsiella pneumoniae*, and *Pantoea unclassified*) belonging to Proteobacteria were enriched in the CAS group, and three species (i.e., *Bacteroides sp 3_1_19, P. unclassified*, and *Prevotella copri*) belonging to Bacteroidetes were enriched in healthy controls.

#### Associations Between the Gut Microbiome and Clinical Characteristics

In this study, we divided clinical characteristics into risk factors and inflammatory markers and used Spearman's correlation analysis to explore their correlations with species abundance. We found that most gut microbes enriched in the CAS group had significant positive correlations with clinical characteristics, whereas those enriched in healthy controls had significant negative correlations with clinical characteristics, excluding HDL-C. More importantly, species more frequently had negative correlations with risk factors than with inflammatory markers (Figure 3B, Supplementary Table 3). Additionally, among the 11 risk factors, BMI had the largest number of correlations with bacterial species (*n* = 9, *p* < 0.05), followed by HDL-C (*n* = 8, *p* < 0.05), TG (*n* = 7, *p* < 0.05), HbA1c (*n* = 6, *p* < 0.05), TC (*n* = 5, *p* < 0.05), LDL-C (*n* = 4, *p* < 0.05), and FBG (*n* = 4, *p* < 0.05). Among the 31 species, *M. elsdenii* had the largest number of correlations with clinical characteristics, followed by *B. sp 3_1_19* (*n* = 6, *p* < 0.05), *Bacteroides coprophilus* (*n* = 5, *p* < 0.05), *G. haemolysans* (*n* = 4, *p* < 0.05), *A.defectiva* (*n* = 4, *p* < 0.05), *P. unclassified* (*n* = 4, *p* < 0.05), and *Actinomyces massiliensis* (*n* = 4, *p* < 0.05; Figure 3C), whereas only *A. massiliensis* was enriched in healthy controls.

### Functional Shifts Linked to the Microbiome in the CAS and Control Groups

#### Functional Shifts in the Microbiome Characteristics of Different Subjects

**Figure 4:**
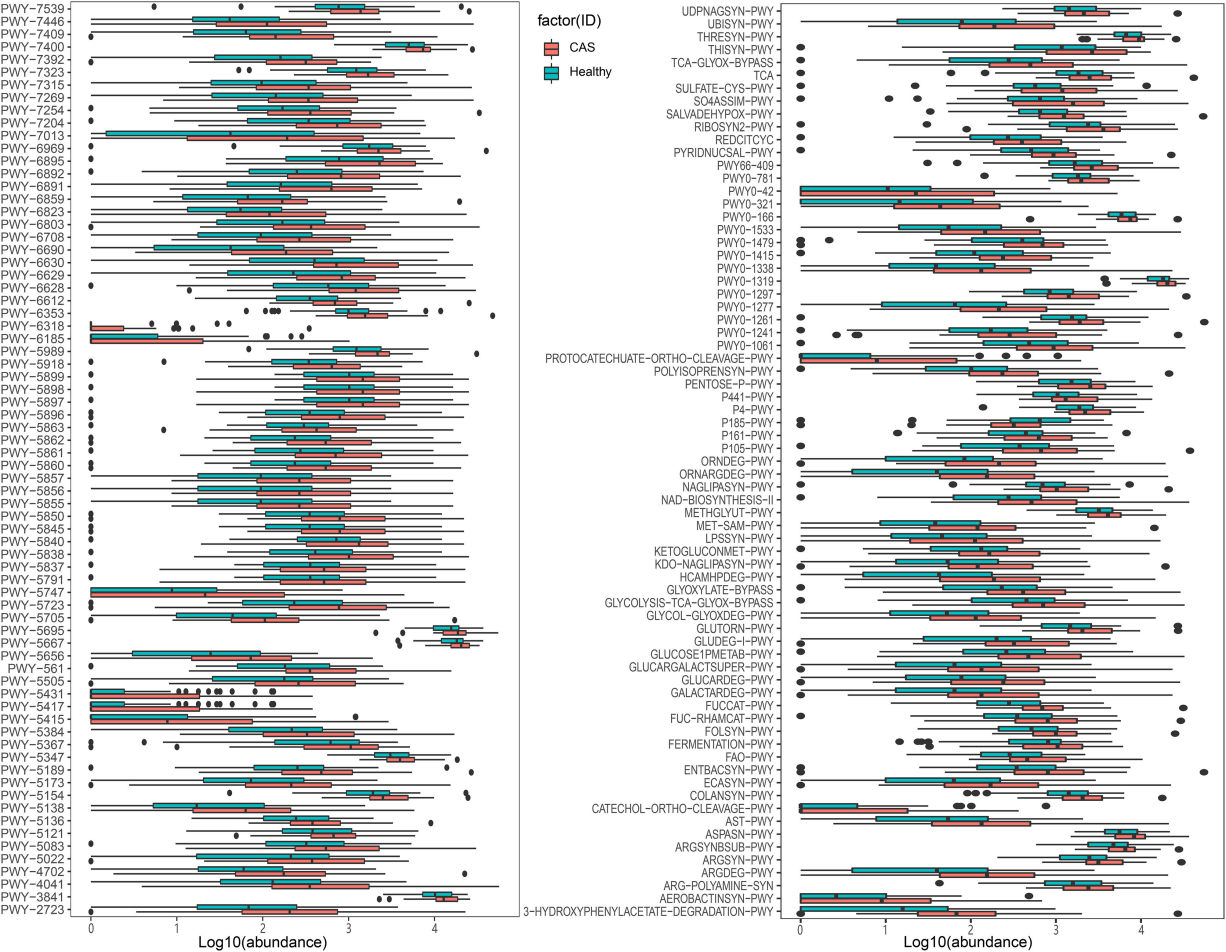
MetaCyc pathways with differences in abundance between the healthy control and carotid arteriosclerosis (CAS) groups (*p* < 0.05, Wilcoxon's rank-sum test).

We further used the MetaCyc pathway database from HUMAnN2 to construct the functional profiles with 494 pathways for each sample. We compared differences inpathway abundance and identified 145 different pathways (Wilcoxon's rank-sum test; *p* < 0.05). After removing the pathways with low occurrence and abundance, 143 pathways with significantly different abundance remained (Supplementary Table 4, Figure 4). In total, 142 pathways were enriched in the CAS group, whereas only one pathway was enriched in the control group. The pathway enriched in healthy controls was formaldehyde assimilation III (*P185-PWY*, metabolism to 3-phospho-D-glycerate), in which formaldehyde is a central metabolite in the generation of TMAO from TMA (*PWY-6968*, trimethylamine degradation).

Among the 142 pathways enriched in the CAS group, 36 were responsible for the biosynthesis of cofactors, carriers, and vitamins; 23 were related to energy generation [tricarboxylic acid cycle (TCA)] and carbohydrate degradation, utilization, and assimilation (secondary metabolite, propanoate, D-glucarate, and sugar); 15 were involved in the biosynthesis of L-arginine, L-ornithine, L-methionine, L-glutamine, L-aspartate, and L-asparagine; 12 were involved in carbohydrate, fatty acid, and lipid biosynthesis; 11 were involved in the biosynthesis of menaquinol and ubiquinol; and five and four were involved in fermentation to short-chain fatty acids (SCFAs) and lipopolysaccharide (LPS) biosynthesis (dTDP-sugar, GDP-sugar, fatty acid, and lipid biosynthesis), respectively. In addition, compared with the findings in healthy controls, patients with CAS had obvious differences in the *PWY-6318* (L-phenylalanine degradation), *PWY-5431* (aromatic compound degradation), *PWY-5415* (catechol degradation), *PWY-5417* (catechol degradation), and *protocatechuate-ortho-cleavage-PWY* pathways (protocatechuate degradation), and the metabolites of these pathways are involved in the TCA cycle.

#### Microbial Pathways Correlated With a Panel of Clinical Characteristics

**Figure 5:**
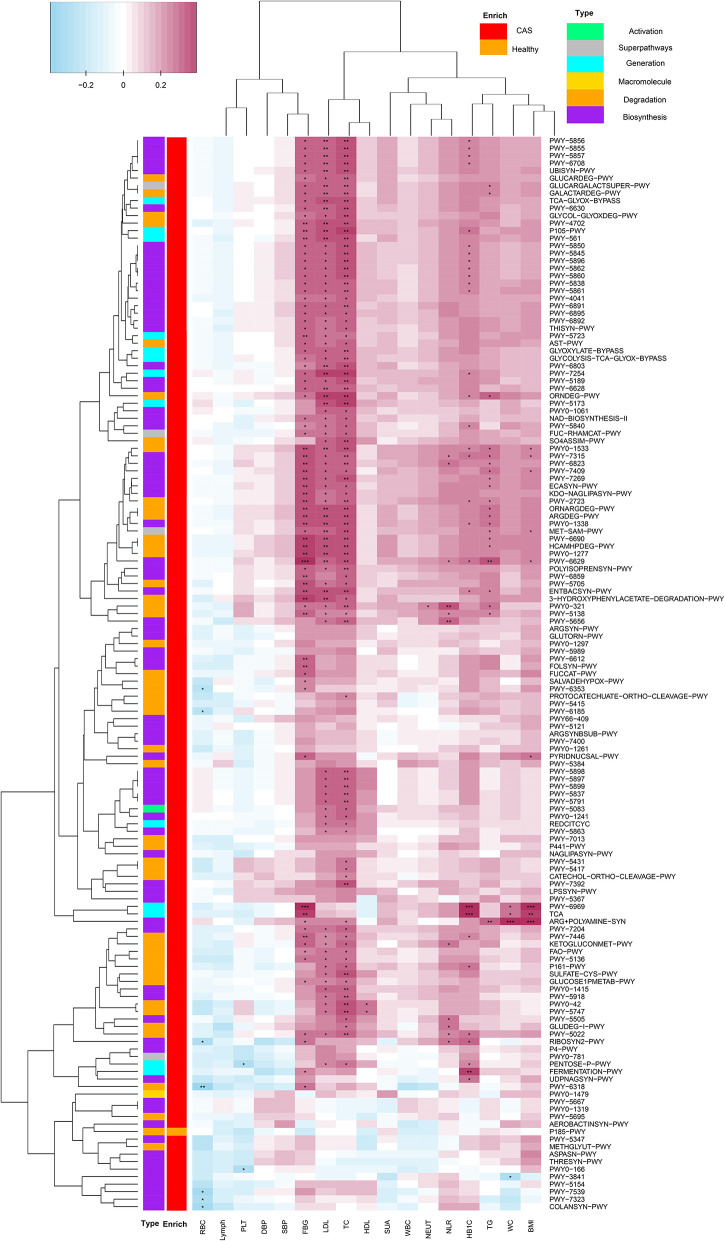
Spearman's correlation matrix for carotid arteriosclerosis-associated pathways and clinical characteristics. Cell color indicates the correlation type (pink: positive, blue: negative). *, **, and ***denote *p* < 0.05, *p* < 0.01, and *p* < 0.001, respectively.

We combined the results of the significantly different MetaCyc pathways and clinical characteristics for Spearman's correlation analyses (*p* < 0.05). Heatmaps were constructed with Spearman's correlations (Figure 5, Supplementary Table 5). Obviously, almost all pathways had positive correlations with clinical characteristics, whereas only a few pathways had negative correlations with inflammatory markers, such as RBC, PLT, and lymph. Importantly, TC, LDL-C, and FBG had significant positive correlations with most pathways enriched in the CAS group, as did TG, HbA1c, NLR, and BMI. Conversely, *P185-PWY*, which was enriched in healthy controls, had negative correlations with most clinical characteristics. It was surprising that only TC and LDL-C had significant positive correlations with menaquinol, L-glutamate, and L-glutamine biosynthesis. In summary, the results suggested that pathways associated with CAS might potentially contribute to the biosynthesis and degradation of lipids and carbohydrates.

#### Microbial Pathways Correlated With a Panel of Microbial Species

**Figure 6:**
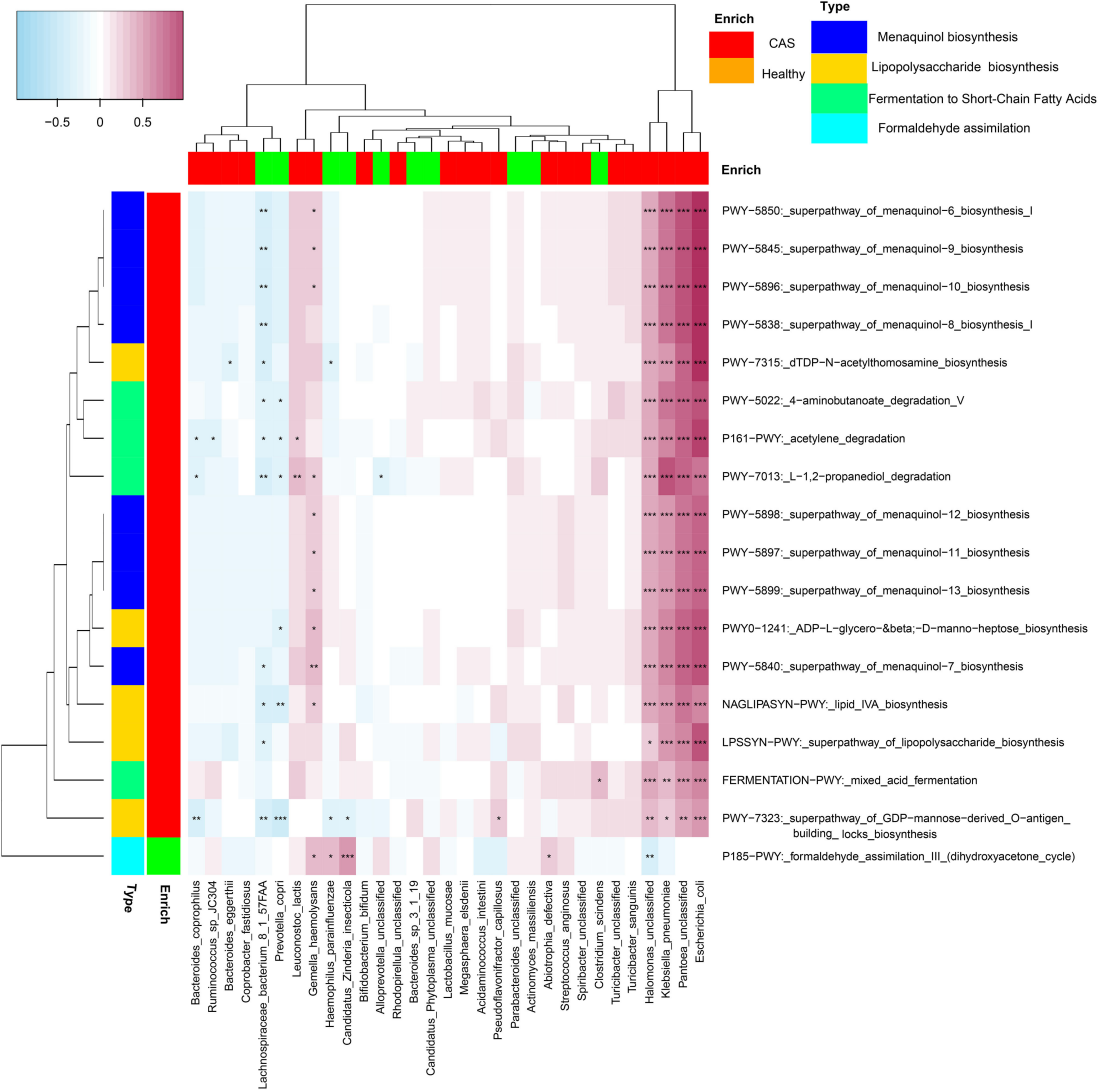
Spearman's correlation matrix for microbial pathways and species. The cell color indicates the correlation type (pink: positive, blue: negative). *, **, and ***denote *p* < 0.05, *p* < 0.01, and *p* < 0.001, respectively.

We selected four categories of microbial pathways, namely, formaldehyde assimilation (enriched in healthy controls), fermentation to SCFA, LPS biosynthesis, and menaquinol biosynthesis (enriched in patients with CAS) and analyzed the correlations between microbial pathways and species using Spearman's correlation analysis (*p* < 0.05). Heatmaps were constructed using Spearman's correlation coefficient (Figure 6, Supplementary Table 6). Interestingly, *E. coli, H. unclassified, K. pneumoniae, and P. unclassified* (enriched in the CAS group) had significantly stronger contributions to pathways responsible for SCFA, LPS, and menaquinol biosynthesis. In particular, *E. coli* had the largest correlation with all pathways enriched in the CAS group from mixed acid fermentation (*r* = 0.521) to menaquinol biosynthesis (*r* = 0.925). Additionally, most microbial species enriched in healthy controls had negative correlations with the pathways enriched in the CAS group and positive correlations with *P185-PWY*. Lastly, for *P185-PWY, Candidatus Zinderia insecticola*, which was enriched in healthy controls, had the greatest significant positive correlation (*r* = 0.470), whereas *H. unclassified*, which was enriched in the CAS group, had a significant negative correlation (*r* = −0.307).

## Discussion

Atherosclerosis is a chronic inflammatory disease that can lead unwittingly to several serious complications such as blood clots, heart attack, stroke, or heart failure; thus, early prevention and treatment are crucial (25). As is well known, the atherosclerotic disease is linked to several risk factors, such as obesity, type 2 diabetes, hyperlipidemia, hypertension, and chronic systemic inflammation (8). In our results, the CAS group exhibited significant differences in risk factors (WC, BMI, DBP, SBP, FBG, HbA1c, TC, TG, LDL-C, and SUA) and inflammatory markers (WBC and NEUT) compared with healthy controls. At present, the therapeutic modalities for CVD and CAS mainly include drugs, surgery, and the control of risk factors (26, 27). However, long-term drug treatment is unfeasible and potentially linked to severe adverse effects, and the compliance with strict diets and exercise in elderly patients is low. Interestingly, there is growing evidence that the human gut microbiome may affect the development and progression of atherosclerosis, thereby providing a new view to exploring the treatment of CAS. Thus, we analyzed the characteristics and correlations of gut microbes with microbial metabolic pathways in Chinese patients with CAS.

We found that Bacteroidetes, Firmicutes, Proteobacteria, and Actinobacteriawere the main flora in both groups, and the abundance of Bacteroideteswas higher in the CAS group than in the control group in prior studies (12). Furthermore, 31 species had significant differences in abundance in both groups, with 21 species enriched in the CAS group and 10 species enriched in the control group. Among the 31 species, seven species had a higher abundance than other species, namely, *Bacteroides eggerthii, E. coli*, and *K. pneumoniae* in the CAS group and *P. unclassified, P. copri, B. sp 3_1_19*, and *Haemophilus parainfluenzae* in the control group. Additionally, we identified risk factors and inflammatory markers with positive correlations with most species enriched in the CAS group, whereas negative correlations were observed with the species enriched in healthy subjects. A previous study about coronary atherosclerosis obtained similar results, namely, the enrichment of *Ruminococcus, E. coli*, and *Streptococcus* in the CAS group and *P. copri, Bacteroides*, and *Parabacteroides* in the control group (15). In addition, previous studies revealed that Ruminococcaceae in the CAS group and *Prevotella* in the control group had a close relationship with the level of TMAO production (28, 29).

In previous studies, TMAO maintained the cell volume in water-stressed organisms and tissues and enhanced protein stability (and favor protein folding) by suppressing the activity of the actomyosin motor in muscular proteins (30). Recently, many studies indicated that the circulating concentrations of TMAO were elevated in patients with CVD, kidney disease, type 2 diabetes mellitus, and cancer, which can promote the development of atherosclerosis (31). TMAO can be obtained from several sources. First, dietary sources, namely, choline, phosphatidylcholine, L-carnitine, and other methylamine-containing nutrients provide substrates for the microbiota-mediated production of TMA, which then enters the portal circulation followed by its conversion by the hepatic flavin-containing monooxygenase family of enzymes into TMAO (32, 33). In our study, some species (*E. coli* and *Halomonas*), the metabolic pathways of menaquinol biosynthesis (PWY-5838, PWY-5840, PWY-5845, and PWY-5899), and TMAO electron transfer (PWY0-1347 and PWY0-1578) were enriched in the CAS group. In addition, these pathways had significant positive correlations with the abundance of *E. coli* and *Halomonas*. *E. coli* carries H (2) uptake hydrogenases (hydrogenase 1 and hydrogenase 2) and two TMAO reductases (the inducible enzyme encoded by torA and a second TMAO reductase encoded by tor Z) (34). *E. coli* and *Halomonas* are involved menaquinol biosynthesis, which could be conducive to TMAO reduction (35, 36). Thus, TMAO might always be involved in the cyclic process of synthesis and reduction in patients with CAS, elevating the circulating concentrations of TMAO and increasing the risk of CAS.

In healthy controls, the P185-PWY pathway (formaldehyde assimilation III) was identified. The key step of this pathway is the transfer of a glycolaldehyde group from D-xylulose 5-phosphate to formaldehyde, forming dihydroxyacetone and D-glyceraldehyde 3-phosphateunder the action of microbial species belonging to *Pichia* and *Candida* (37). In addition, in the TMAO production pathway (PWY-6968 pathway), the trimethylamine-oxide aldolase catalyzes the non-oxidative, nonhydrolytic cleavage of TMAO to dimethylamine and formaldehyde (38). Thus, formaldehyde may act as an important molecule for lowering the circulating concentrations of TMAO. In previous studies, converting TMA into methane and changing the activity of FMO3 were deemed the most effective strategies for reducing TMAO levels (39, 40).

In addition, our results indicate that some gram-negative bacteria enriched in the CAS group, such as *E. coli* and *K. pneumoniae*, have strong positive correlations with LPS biosynthetic pathways, resulting in increased plasma LPS levels. LPS can promote the recruitment of adaptor proteins (41), induce a low-grade inflammatory state, and aggravate the progression of atherosclerosis (12, 42). Thus, in our results, most LPS biosynthetic pathways were positively correlated with WBC, PLT, NEUT, and NLR. However, *H. parainfluenzae* and *P. copri*, which wereenriched in healthy controls, had negative correlations with LPS biosynthetic pathways, thereby decreasing LPS levels and reducing the disease risk of CAS.

## Conclusion

In this study, we identified metagenomic associations between the gut microbiome and CAS. Compared with the findings in healthy controls, some risk factors and inflammatory markers had strongly positive correlations with microbial species and pathways that were enriched in patients with CAS, suggesting an intrinsic connection between the gut microbiome and CAS. Additionally, specific microbial species and pathways that were closely associated with TMAO levels and LPS biosynthesis might represent treatment targets for new therapies for metabolic disorders. In future research, in consideration of the stability of CAS, we will divide the carotid plaque into different groups to identify specific microbial species and pathways. Additionally, we will perform animal experiments to verify the results.

## Data Availability Statement

According to national legislation/guidelines, specifically the Administrative Regulations of the People's Republic of China on Human Genetic Resources (http://www.gov.cn/zhengce/content/2019-06/10/content_5398829.htm, http://english.www.gov.cn/policies/latest_releases/2019/06/10/content_281476708945462.htm), no additional raw data is available at this time. Data of this project can be accessed after an approval application to the China National Genebank (CNGB, https://db.cngb.org/cnsa/). Please refer to https://db.cngb.org/, or email: CNGBdb@cngb.org for detailed application guidance. The accession code CNP0002210 should be included in the application.

## Ethics Statement

The studies involving human participants were reviewed and approved by the Ethics Committee from the First Affiliated Hospital of Zhengzhou University. The patients/participants provided their written informed consent to participate in this study.

## Author Contributions

SD and QQ: conceptualization. JC, SY, and AL: methodology and formal analysis. YY, TL, LW, and XG: resources. JC: writing—original draft preparation. SD and QQ: writing—review and editing. JC: visualization. All the authors have read and agreed to submit the manuscript.

## Funding

This study was equally funded by the National Natural Science Foundation of China (Grant No. 72101236), the Henan Province Key Scientific Research Projects of Universities (Grant No. 21A320035), the Henan Province Youth Talent Promotion Project (Grant No. 2021HYTP052), the Henan Province Medical Science and Technology Research Plan (LHGJ20200279), and the Chinese National Science and Technology Major Project 2018ZX10305410.

## Conflict of Interest

The authors declare that the research was conducted in the absence of any commercial or financial relationships that could be construed as a potential conflict of interest.

## Publisher's Note

All claims expressed in this article are solely those of the authors and do not necessarily represent those of their affiliated organizations, or those of the publisher, the editors and the reviewers. Any product that may be evaluated in this article, or claim that may be made by its manufacturer, is not guaranteed or endorsed by the publisher.

## Supplementary Material

Supplementary Tables 1-7 The detailed experimental results between the CAS group and healthy controls.
